# Characterization of walnut *JrWOX11* and its overexpression provide insights into adventitious root formation and development and abiotic stress tolerance

**DOI:** 10.3389/fpls.2022.951737

**Published:** 2022-09-06

**Authors:** Yingying Chang, Xiaobo Song, Mingjun Li, Qixiang Zhang, Pu Zhang, Xiashuo Lei, Dong Pei

**Affiliations:** ^1^Engineering Laboratory of Green Medicinal Material Biotechnology of Henan Province, Engineering Technology Research Center of Nursing and Utilization of Genuine Chinese Crude Drugs of Henan Province, College of Life Science, Henan Normal University, Xinxiang, China; ^2^State Key Laboratory of Tree Genetics and Breeding, Key Laboratory of Tree Breeding and Cultivation of the State Forestry and Grassland Administration, Research Institute of Forestry, Chinese Academy of Forestry, Beijing, China; ^3^The Nurturing Station for the State Key Laboratory of Subtropical Silviculture, School of Forestry and Biotechnology, Zhejiang A&F University, Lin’an, China

**Keywords:** *JrWOX11*, overexpression, adventitious root formation, abiotic stress tolerance, motif-”GGAIQY”

## Abstract

The well-developed root system enables plant survival under various environmental stresses. *WUSCHEL-RELATED HOMEOBOX GENE 11* (*WOX11*) plays a critical role in adventitious root formation and development in rice, *Arabidopsis*, and easy-to-root tree poplar. However, in difficult-to-root trees, the knowledge of *WOX11* during adventitious root formation and development remains scarce. In this study, the *JrWOX11* gene was isolated from a difficult-to-root tree walnut and heterologously expressed in the “84K” poplar. The results showed that *JrWOX11* contained a similar structure and sequence to the homologous genes in rice, *Arabidopsis*, and poplar, but had different numbers and types of motifs and *cis*-elements. JrWOX11 lacked the motif GGAIQY compared to that in easy-to-root trees. In addition, *JrWOX11* expression was induced by ABA, PEG, and NaCl treatments. Overexpression of *JrWOX11* in poplar promoted root initiation and significantly increased adventitious root (ARs) number, lateral roots (LRs) number, and root hair (RH) length. Furthermore, the aboveground biomass was notably increased under NaCl and PEG treatments in transgenic plants. When NaCl and PEG were removed, the survival rate, aerial shoot development, and *de novo* root organogenesis were also markedly enhanced in transgenic shoot cuttings. The study provides valuable information on the differences between *JrWOX11* and the homologous genes in rice, *Arabidopsis*, and poplar, and supports the critical role of *JrWOX11* in the formation of AR and tolerance to salt and osmotic stresses.

## Key message

– WOX11 in walnut lacks the motif GGAIQY compared to that in easy-to-root trees.
– *JrWOX11* overexpression enhanced stress tolerance by promoting the rooting capacity and regulating root system architecture.

## Introduction

Walnut (*Juglans regia* L.) is the most widespread nut tree species globally and is thus economically important (Zhang et al., 2020). It is also a difficult-to-root tree (Pei and Gu, 2002). In difficult-to-root trees, such as some *Eucalyptus*, *Pinus*, *Juglans*, *Carya*, *Quercus*, and *Castanea* (Basheer-Salimia, 2007; Amissah et al., 2008; Liu H. et al., 2018), the capacity of cuttings to form AR is relatively low, especially when the mature plant organs, such as shoots, were used as explants (Pijut et al., 2011). This is a significant limitation in the clonal propagation of commercial germplasm in difficult-to-root trees.

To overcome the problems associated with the loss of, or reduced, the competence of difficult-to-root trees to form AR, extensive studies have been conducted (Klerk, 2002; Gonin et al., 2019). For easy-to-root plants, a high auxin concentration above the cut site (due to auxin biosynthesis and auxin flow induced by wounding) is generally enough for adventitious root formation (Da Costa et al., 2013; Zhang et al., 2019). The detached leaves of *Arabidopsis thaliana* placed on a B5 medium initiate adventitious root formation at the cut site without any exogenous plant hormones (Chen et al., 2014). By contrast, in difficult-to-root trees, rejuvenation treatment and exogenous auxin are two prerequisites for rooting (Pei et al., 2004). Some previous studies have suggested that the rejuvenation treatment could reduce the thickness and density of sclerenchyma between the cortex and phloem, which is beneficial to rooting (Maynard and Bassuk, 1990; Liu H. et al., 2018). Rejuvenation treatment also induced the expression of the *WOX11* gene in walnut (Chang et al., 2020). Given the role *WOX11/12* plays in the process of adventitious root formation in difficult-to-root trees, it is reasonable to hypothesize that there might be some key differences in *WOX11/12* between easy-to-root plants and difficult-to-root trees.

The *WOX11/12* gene is one member of the intermediate clade in the *WUSCHEL*-related homeobox (*WOX*) family (Liu and Xu, 2018). In *Arabidopsis*, the expression of *AtWOX11* and *AtWOX12* can be induced by auxin, and AtWOX11 acts redundantly with AtWOX12 to stimulate cell fate transition to root founder cells (Liu J. et al., 2014). In rice, *OsWOX11*, *OsWOX12A*, and *OsWOX12B* could respond rapidly to auxin, cytokinin, and abiotic stress stimuli of drought, salt, and cold (Cheng et al., 2014; Jiang et al., 2017). Overexpression of *OsWOX11* in rice improves crown root emergence and growth, with the well-developed root system enabling rice survival under drought stress (Zhao et al., 2009; Cheng et al., 2016). In easy-to-root tree species poplar, such as *P. tomentosa*, “Nan-lin895” (*P. deltoides* × *P. euramericana*) and “84K” poplar (*P. alba* × *P. glandulosa*), *WOX11/12s* not only promoted adventitious root formation and increased the number of AR on the cuttings but also induced ectopic roots in the aerial parts of transgenic poplars (Liu B. et al., 2014; Xu et al., 2015; Wang et al., 2020). A new study has also reported that *PagWOX11/12a* enhanced the plant response to salt stress through the control of redox metabolic pathways (Wang et al., 2021). Despite the functional importance of WOX11/12 protein in adventitious root formation and development and stress resistance in easy-to-root plants, the current knowledge regarding the *WOX11/12* gene in difficult-to-root trees is scarce.

In this work, the *JrWOX11* gene was cloned from hybrid walnut “Zhongningsheng” (*J. hindsii* × *J. regia, “*ZNS”). The *JrWOX11* gene contained a similar structure and conserved domain sequence to the homologous genes in rice, *Arabidopsis*, and poplar, but had different numbers and types of *cis*-elements. Expression analysis showed that the *JrWOX11* transcript was expressed at significantly higher levels in roots than in other tissues and was induced by abscisic acid (ABA), salt (NaCl), and polyethylene glycol (PEG). Additionally, the overexpression of *JrWOX11* promoted adventitious root formation in poplar. The AR number, LR number, LR length, and RH length were also increased. Moreover, the tolerance to salt and osmotic stress (NaCl and PEG) in transgenic poplar (*P. alba* × *P. glandulosa* clone “84K”) plants was enhanced. Our results demonstrated that *JrWOX11* plays a crucial role in adventitious root formation and confers salt and osmotic stress tolerance in walnut.

## Materials and methods

### Plant materials

Hybrid poplar (*P. alba* × *P. glandulosa*) clone “84K” was used for transformation. The sterile seedlings of “84K” were cultured on half MS medium supplemented with 0.05 mg⋅L^–1^ of indole-3-butyric acid (IBA) and 0.02 mg⋅L^–1^ of naphthylacetic acid (NAA) in the tissue culture lab of the China Academy of Forestry, Beijing, China. The growth conditions were kept at 23∼25°C with 16 h light/8 h dark photoperiod. The shoots were subcultured on the same amount of fresh medium every 4 weeks.

Tobacco plants (*Nicotiana benthamiana*) were grown in pots under an 8 h day/16 h night photoperiod at 23°C. After ∼5 weeks, when 7∼10 tobacco leaves became expanded, the top leaves (third∼fifth) were used for transient expression.

The 1-year-old seedlings of hybrid walnut “ZNS,” a walnut stock cultivar characterized by high yields, good adaptability, and high graft compatibility, were cultured in the greenhouse of the China Academy of Forestry, Beijing, China. The growth condition was kept at ∼25°C with a humidity of 50∼60%.

### RNA isolation, DNA isolation, gene cloning, and vector construction

An RNeasy Plant Mini Kit and an RNase-free DNase I kit (Qiagen, Hilden, Germany) were used to extract total RNA from the rejuvenated stem of “ZNS.” Complementary DNA (cDNA) synthesized by the SuperScript II reverse transcriptase (Thermo Fisher Scientific, Vilnius, Lithuania) was used as the template for cloning the coding sequence (CDS) of the *JrWOX11* gene. The putative sequence of *WOX11* online (GenBank accession no. XM_018977839) was used for primers design. The PCR product was cloned into pDONR222 and then recombined into the pMDC32 vector and pEarleyGate104 vector (ABRC stock DB3-686) to produce *35S:JrWOX11* and *35S:YFP-JrWOX11* constructs, respectively. Both of the above constructs were introduced into *Agrobacterium tumefaciens* strain GV3101 by electroporation.

Genomic DNA was extracted from the leaves of 1-year-old seedlings of “ZNS” walnut *via* a Plant DNA Isolation Reagent kit (Takara, Kyoto, Japan) and was used as the template for cloning *JrWOX11* promoter (-2,000 bp to + 206 bp). The PCR product was cloned into the pCAMBI1031 vector. All the above gene-specific primers are shown in Supplementary Table 1.

### The analysis of the *WOX11* gene

The protein sequences of *WOX11/12* homologous genes in monocotyledon *Oryza sativa*, dicotyledonous herb *Arabidopsis thaliana*, easy-to-root trees *Populus trichocarpa*, *P. tomentosa*, “Nan-lin895” (*P. deltoides* × *P. euramericana*), “84K” poplar (*P. alba* × *P. glandulosa*), *Salix purpurea*, *Malus domestica*, *Morus notabilis*, *Durio zibethinus*, and difficult-to-root trees *J. regia*, *Carya illinoinensis*, *Quercus suber*, *Quercus lobata*, and *Castanea mollissima* were downloaded from NCBI and phytozome website. The motif enrichment and sequence distances compared to JrWOX11 protein were analyzed by MegAlign software (DNASTAR. Madison, WI, United States) and MEME v4.9.0 online software.^1^ The gff3 files of *JrWOX11* (refer to XM_018977839) in walnut and *OsWOX11*, *OsWOX12a*, *OsWOX12b*, *AtWOX11*, *AtWOX12*, *PrtWOX11/12a*, and *PrtWOX11/12b* in model plants were used to analyze gene structure by Gene Structure Display Server.^2^

The upstream regions (2.0 kb) of the translation initiation sites (ATG) of *WOX11/12* homologous genes in *O. sativa*, *A. thaliana*, and *P. trichocarpa* were used as promoter fragments for the *cis*-elements analysis compared with that of *JrWOX11* gene using the program PlantCARE online.^3^

### Subcellular localization of *JrWOX11*

To evaluate *JrWOX11* localization within cells, we utilized a *35S:YFP-JrWOX11* construct, such that the *JrWOX11* coding region was fused to the YFP C-terminus. The *A. tumefaciens* GV3101 colony carrying the *35S:YFP* construct, RFP-H2B (a nucleus localization signal marker tagged with RFP) and OsSP1-RFP (a plasma membrane localization marker) were individually cultured to OD_600_ = 0.4–0.6, re-suspended with the 10 mM EMS, 10 mM MgCl_2_ (pH 5.8) and 150 μM acetosyringone to OD_600_ = 0.2 (1:1:1 v/v/v), and co-infiltrated into *N. benthamiana* leaves as a control. The *A. tumefaciens* GV3101 cells containing the *35S:YFP-JrWOX11* construct and RFP-H2B were individually cultured, re-suspended, mixed, and co-infiltrated into *N. benthamiana* leaves as described above to clarify the subcellular localization of *JrWOX11*. After about 48∼56 h incubation at 23°C under an 8 h day/16 h night photoperiod, *N. benthamiana* leaves were collected to observe the excitation of YFP and RFP using an LSM 510 confocal laser scanning microscope (Carl Zeiss AG, Oberkochen, Germany) with fluorescence imaging at 488 and 561 nm, respectively, and emission being captured between 500 and 550 nm.

### Tissue-specific and inducible expression of *JrWOX11* treated with abscisic acid and salt and osmotic stress

To reveal expression profiles of *JrWOX11* gene in different organs, including roots (R), stem (S), leaf (L), female flower (FF), immature fruit (IF), and zygotic embryo (ZE), they were collected from 23-year-old “ZNS” trees from Luoning county, Henan Province. Six samples of each tissue from the same tree were mixed as one biological replicate, and three replicate trees were sampled. All samples were snap-frozen in liquid nitrogen and stored at −80°C for the expression analysis of *JrWOX11* as described in the previous study (Chang et al., 2020).

To investigate the expression profiles of *JrWOX11* under salt and osmotic stress, the 1-year-old “ZNS” seedlings were irrigated with 200 mM NaCl and 10% (w/v) PEG 6000 solution, and the roots were collected after seven treatment durations (0, 1, 3, 6, 9, 12, and 24 h). For ABA treatment, the top fourth–sixth walnut leaves were sprayed with 100 μM ABA and collected after 0, 1, 3, 6, 9, 12, and 24 h. Ten leaf sections were used for each treatment, and experiments were repeated three times. All samples were snap-frozen in liquid nitrogen and stored at −80°C for the expression analysis of *JrWOX11*.

Total RNA was isolated using the method described above (Section “RNA isolation, DNA isolation, gene cloning, and vector construction”). The synthesized cDNA was diluted 10-fold with ddH_2_O to serve as a template for qRT-PCR on a Roche LightCycler 480 (Roche Applied Science, Penzberg, Upper Bavaria, Germany). Expression levels were normalized relative to the control (*GAPDH*) using the 2−△△*Ct* method. The qRT-PCR primers are shown in Supplementary Table 1.

### Stable expression of *JrWOX11* in transgenic hybrid poplar “84K”

An *A. tumefaciens* strain GV3101 containing the *35S:JrWOX11* construct was used for the transformation of poplar “84K” as described previously (Liu B. et al., 2014; Li et al., 2017). The discs from the top fourth–fifth leaves from 4-week-old “84K” were notched and incubated with *A. tumefaciens* GV3101 cells containing the *35S:JrWOX11* construct for about 3 days. The hygromycin-resistant shoots were obtained from the leaf explants after 2–4 weeks on the MS medium supplemented with 0.5 mg⋅L^–1^ of 6-BA, 0.05 mg⋅L^–1^ of NAA, 3 mg⋅L^–1^ of hygromycin, and 200 mg⋅L^–1^ of timentin. Roots were induced on root induction medium (RIM: 1/2 MS + 0.05 mg⋅L^–1^ of IBA + 0.02 mg⋅L^–1^ of NAA) + 3 mg⋅L^–1^ of hygromycin. The resistant plants were detected by qRT-PCR of the *JrWOX11* gene expression. The plants with high expression levels of the *JrWOX11* gene were subcultured for later experiments.

### Plant phenotype determination

To determine the role of the *JrWOX11* gene in root formation, a stereo microscope (Leica, Wetzlar, Germany) was used to observe the stem samples from the control and transgenic (OE6#) “84K” after 0, 1, 2, 3, 4, 5, and 6 days on RIM. Cross-sections (30 μm thick) of the stem base were prepared with a VT-1000S vibrating blade microtome (VT-1000S, Leica, Wetzlar, Germany) and stained with 2% w/v toluidine blue O solution for visualizing root primordia under a BX51 digital microscope (Olympus, Tokyo, Japan). Five seedlings of each line per replicate were used, and two independent experiments were performed.

To analyze the rooting index, including the AR number, LR number, AR length, LR length, and root hair (RH) length, the shoots (about 2.5 cm high) were cut from 4-week-old non-transgenic “84K” (CK) and transgenic seedlings (OE#3 and OE#6) and moved to RIM under 16 h light/8 h dark for about 2 and 4 weeks, respectively. Ten individuals per replicate were used for each line, and three independent experiments were performed.

### Analysis of salt and osmotic stress tolerance

To evaluate salt and osmotic stress tolerance, 2-week-old non-transgenic “84K” (CK) and transgenic (OE3# and OE6#) surface-sterilized plants were grown on RIM supplemented with 0 (control) or 200 mM NaCl (salt treatment) or 5% w/v PEG 6000. Three weeks later, the plant height and fresh weight (FW) were measured, and the dry weight (DW) was determined after oven drying at 80°C for 72 h. Six biological replicates of each line per treatment were used, and two independent experiments were performed.

To examine the rooting capacity under salt and osmotic stress, shoot segments (about 2.5 cm high) of 4-week-old non-transgenic “84K” (CK) and transgenic (OE3# and OE6#) surface-sterilized plants were cultivated on RIM supplemented with 0 (control) or 200 mM NaCl (salt treatment) or 5% w/v PEG 6000 for 4 weeks and then transferred to RIM for 2 weeks. The phenotypes of the root systems were photographed every week, and the survival rate, the number of axillary buds regenerated, and the number of ARs were quantified. Six biological replicates of each line per treatment were used, and two independent experiments were performed.

### Statistical analysis

Significant differences between means of CK and transgenic lines were determined using the SPSS statistical package (version 16.0; SPSS Inc., Chicago, IL, United States) at a significance level of ^∗^*P* < 0.05 and ^∗∗^*P* < 0.01 (Student’s *t*-test).

## Results

### Identification and characterization of *JrWOX11* gene

**FIGURE 1:**
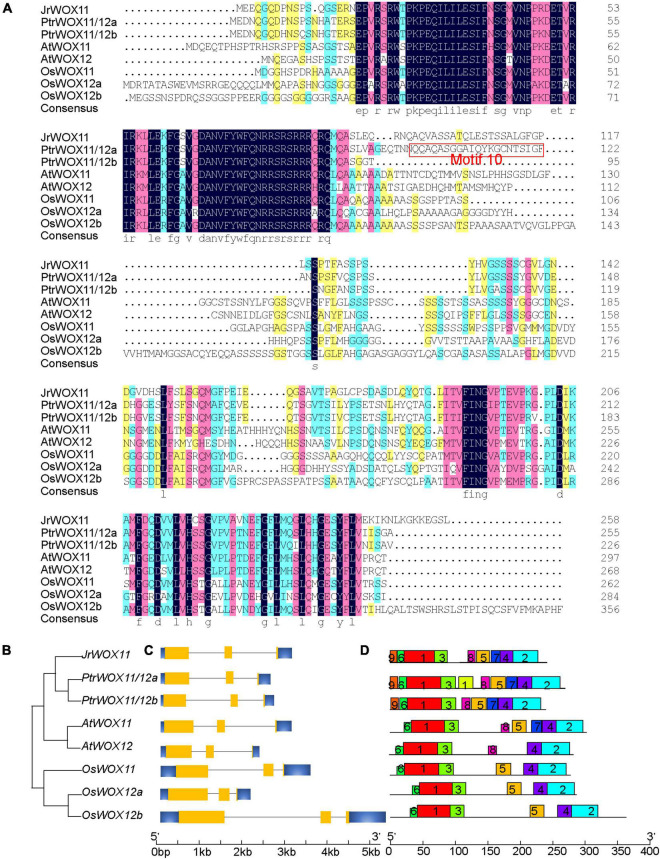
Identification and characterization of cloning of the *JrWOX11* gene. **(A)** Sequence alignment of homologous WOX11/12 proteins. Protein sequences were obtained from NCBI and are shown in Supplementary Table 2. **(B)** Phylogenetic tree based on full-length coding sequences of *WOX11/12* genes. **(C)** Gene structure analysis of *WOX11/12* genes. **(D)** Motifs analysis of WOX11 proteins. MEME software was used to search motifs, and then redraw the map with the software TBtools (Chen et al., 2020). Motif10 was marked with a red box in panel **(A)**.

In “ZNS” walnut, only one *WOX11/12* gene was isolated and submitted to NCBI’s GenBank (*JrWOX11*, NCBI Accession No: ON979687) (Figure 1A). The full-length sequence of *JrWOX11* cDNA is 747 nucleotides and encodes a deduced protein of 248 amino acids (aa) residues, with a predicted molecular mass of 27.05 kDa and a theoretical pI of 5.42 (Supplementary Table 2). However, there were 3, 2, and 2 *WOX11/12* genes in rice, *Arabidopsis*, and *P. trichocarpa*, respectively (Figure 1A) (Zhang et al., 2010; Cheng et al., 2014). Twenty-six WOX11/12 protein sequences from 15 species, namely, two monocotyledons (maize and rice), a dicotyledonous herb (*A. thaliana*), eight easy-to-root trees (*P. trichocarpa*, *P. tomentosa*, Nan-lin895 poplar, “84K” poplar, *S. purpurea*, *Malus domestica, Morus notabilis*, and *D. zibethinus*), and four difficult-to-root trees (*Carya illinoinensis*, *Q. suber*, *Q. lobata*, and *Castanea mollissima*), were downloaded from NCBI and characterized. The protein length ranged from 236 to 356 aa, the pIs from 5.42 to 8.67, and the molecular mass from 25.92 to 36.47 kDa. Multialignment of WOX11/12 protein sequences (by MegAlign software) showed that CiWOX11 (96.37%) in *Carya illinoinensis*, QlWOX11 (82.57%) in *Q. lobata*, QsWOX11 (81.82%) in *Q. suber*, and CmWOX11 (82.23%) in *Castanea mollissima* shared high sequence identity with JrWOX11 in “ZNS” walnut. The WOX11/12 in *Populus*, *S. purpurea*, *Malus domestica*, *Morus notabilis*, and *D. zibethinus* had relatively moderate sequence identities with JrWOX11, ranging from 61.48 to 74.44%. The sequence of WOX11/12 in *Z. mays*, *O. sativa* and *A. thaliana* had relatively low sequence identities with JrWOX11, ranging from 45.22 to 57.01% (Supplementary Table 2). The simplified neighbor-joining (NJ) phylogenetic tree of the related species also showed that JrWOX11 was similar to PtrWOX11/12a and PtrWOX11/12b (Figure 1B).

Gene structure analysis revealed that the homologous *WOX11* genes had the same gene structure, such as three exons and two introns (Figure 1C). Motif enrichment analysis revealed five prominent motifs (Figure 1D). Among them, motif1, motif3, and motif6 were related to the homeodomain, whereas motif2 and motif4 were associated with the C-terminus domain of intermediate clade WOXs (Zhang et al., 2010). Motif7 and motif8 were observed only in dicotyledons, and motif9 was conserved only in the easy-to-root trees. Even though AtWOX11 and AtWOX12 have a similar function in adventitious root formation, motif5 was missing in AtWOX12. Furthermore, motif10 was only found in easy-to-root trees, such as *P. trichocarpa*, *Ptr*; *S. purpurea*, *Sp*; *Malus domestica*, *Md*; *Morus notabilis*, *Mn*; and *D. zibethinus*, *Dz*; but was not present in difficult-to-root trees, such as *J. regia*, *Jr*; *Carya illinoinensis*, *Ci*; *Quercus suber*, *Qs*; *Quercus lobata*, *Ql*, and *Castanea mollissima*, *Cm*. The sequence of motif10 in PtrWOX11/12a was “QQAQASGGAIQYKGCNTSIGF,” among which “GGAIQY” was extremely conserved (Figures 1A,D and Supplementary Figure 2). To sum up, there might be differences in the number of WOX11/12 proteins and the motifs between easy-to-root trees and difficult-to-root trees.

### Analysis of *JrWOX11* promoter sequence

**FIGURE 2:**
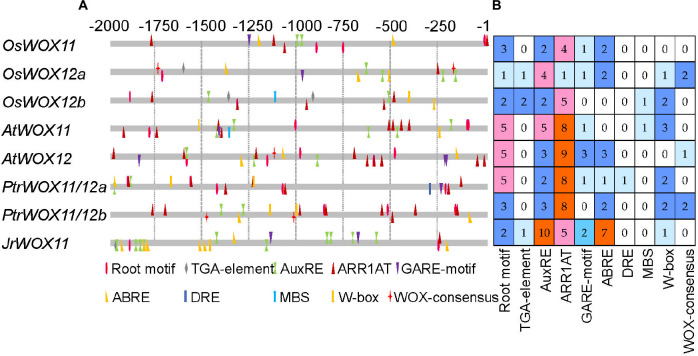
The *cis*-element distribution and number analysis of the promoter region of *WOX11/12* genes in *Oryza sativa*, *Arabidopsis thaliana*, *Populus trichocarpa*, and *Juglans hindsii* × *Juglans regia* cv. “ZNS.” **(A)** The position of the *cis*-element in the upstream 2,000 bp region of *WOX11/12* genes. **(B)** The number of each *cis*-element.

The 2 kb sequence upstream of *WOX11/12s* CDS was used to identify *cis*-acting elements. The results showed that the identified *cis*-elements could be divided into four categories: (1) growth- and development-responsive elements, (2) plant hormone-responsive, (3) abiotic stress-responsive elements, and (4) WOX-consensus motif (Figure 2 and Supplementary Table 3). There were 3, 1, 2, 5, 5, 5, 3, and 2 root-specific elements in the promoter region of *OsWOX11*, *OsWOX12a*, *OsWOX12b*, *AtWOX11*, *AtWOX12*, *PtrWOX11/12a*, *PtrWOX11/12b*, and *JrWOX11*, respectively. Plant hormone-related elements were also identified, such as 2, 5, 4, 5, 3, 2, 3, and 10 auxin-responsive elements (ARFAT and TGA elements), 4, 1, 5, 8, 9, 8, 8, and 5 cytokinin-responsive element (ARR1AT), 1, 1, 0, 1, 3, 1, 0, and 2 gibberellin-responsive element (GARE1OSREP1), and 2, 3, 0, 0, 3, 1, 2, and 7 abscisic acid-responsive element (ABRE), in the promoter region of *OsWOX11*, *OsWOX12a*, *OsWOX12b*, *AtWOX11*, *AtWOX12*, *PtrWOX11/12a*, *PtrWOX11/12b*, and *JrWOX11*, respectively. A few elements responsive to various abiotic stresses were also identified, mainly including DRE, MBS, G-box, and W-box, and the total numbers were 0, 3, 3, 4, 1, 3, 4, and 1 in the above sequence of *WOX* genes. Compared to rice, *Arabidopsi*s, and poplar, the promoter region of *WOX11* in walnut contained fewer root motifs and stress-responsive elements but contained more abscisic acid-responsive elements and AuxRE.

WOX11 is shown to specifically bind to the WOX-consensus “TTAATGG/C” motif (Lohmann et al., 2001; Zhao et al., 2009, 2015; Liu B. et al., 2018). Within the *OsWOX12*, *AtWOX12*, and *PtrWOX11/12b* genes, we identified one or two WOX-consensus motifs common to their promoters. However, walnut did not harbor any *WOX11/WOX12b* genes, suggesting that WOX11-binding genes might differ in different plants (Figure 2 and Supplementary Table 3).

### Subcellular localization of *JrWOX11*

**FIGURE 3:**
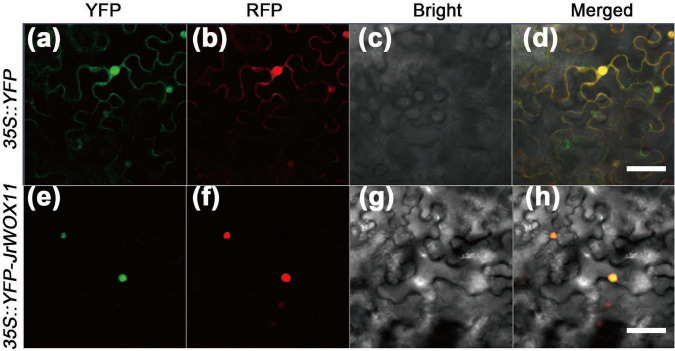
Subcellular localization of JrWO11 protein. 35S::YFP and 35S::YFP-JrWO11 fusion proteins were expressed transiently into *Nicotiana benthamiana* leaf epidermal cells 48∼56 h after vacuum injection. The YFP channel **(a,e)**, the RFP channel **(b,f)**, the bright field **(c,g)**, and the merged images **(d,h)**. Scale bar: 50 μm.

In our previous research, JrWOX11 was predicted to be localized in the nucleus (Chang et al., 2020). To validate this prediction, the *35S:YFP-JrWOX11* construct was transiently co-expressed with the nuclear marker in tobacco leaves. The *35S:YFP* construct mixed with the nucleus and the membrane markers was transformed as a control. The nucleus and plasma membrane were marked by RFP staining. As shown in Figures 3e–h, the 35S:YFP-JrWOX11 fluorescent signals and nucleus marker were observed in the same location, indicating that JrWOX11 is localized in the nucleus. This was consistent with the prediction. By contrast, in cells transformed with the positive control *35S:YFP*, the fusion protein was distributed throughout the cell, including the nucleus and cytoplasm, which were marked by the nuclear and plasma membrane RFP marker staining (Figures 3a–d).

### Tissue-specific and abiotic stress response in the expression of the *JrWOX11* gene

**FIGURE 4:**
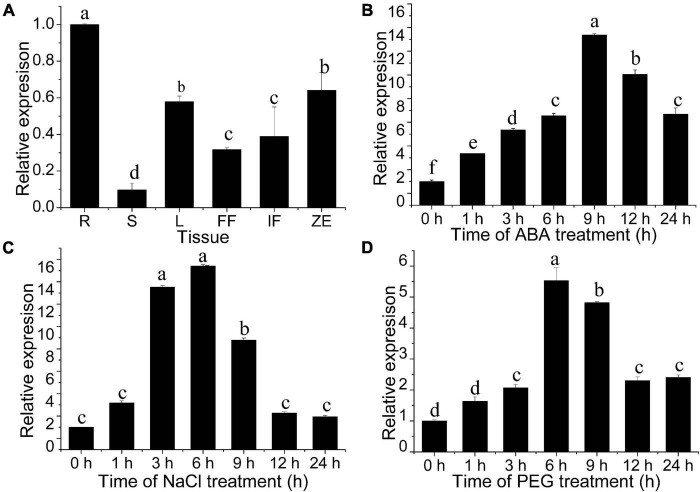
Expression patterns of *JrWOX11* as assessed by qRT-PCR. **(A)** Spatial and temporal expression of *JrWOX11* in different tissues. **(B–D)** Relative expression of *JrWOX11* under 100 μM ABA, salt (200 mM NaCl) and 10% (w/v) PEG 6000 solution. Error bars = SEM. Statistical differences were analyzed using Student’s *t-*test, *n* = 3. Different letters labeled indicate significant differences.

The spatial and temporal expression patterns of the *JrWOX11* gene in different tissues were analyzed using qRT-PCR. As shown in Figure 4A and our previous study (Chang et al., 2020), the *JrWOX11* showed relatively high expression in roots (R), but relatively low expression in stems (S). Previous studies reported that the *WOX11* gene was involved in drought and salt resistance (Cheng et al., 2016; Wang et al., 2018, 2020). Here, the transcription levels of *JrWOX11* in response to ABA, NaCl, and PEG were analyzed. As shown in Figures 4B–D, *JrWOX11* transcription was rapidly induced after treatment with ABA, NaCl, and PEG. The transcription levels increased with prolonged treatments, peaking at 9, 6, and 6 h, respectively, then slightly decreased after that. It could be concluded that the expression of *JrWOX11* is responsive to ABA, NaCl, and PEG.

### Overexpression of *JrWOX11* promoted adventitious root formation in “84K” poplar

**FIGURE 5:**
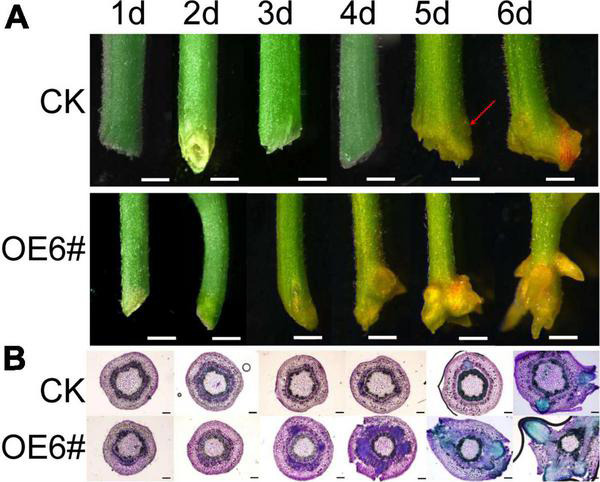
Overexpression of *JrWOX11* promotes root formation. **(A)** The phenotype investigation of the poplar stem base during rooting 1–6 days. Bar = 1 mm. **(B)** The histological investigation of the poplar stem base during rooting 1–6 days. Bar = 200 μm.

The *JrWOX11* had the highest expression in roots, which may play a crucial role in root growth and development. To verify this, *JrWOX11* was transformed into “84K” poplar, resulting in 14 transgenic lines (Supplementary Figure 1). Lines OE3# and OE6# with high expression of JrWOX11 were used in the work reported here. The phenotypes of the stem base of control (CK) and the OE6# line were analyzed by stereo microscopy and sectioning (Figures 5A,B). The results showed that on day 5, small protrusions from the stem base were observed for the first time in control stems, which might have represented a bump of AR primordium. On day 6, the skin of the original protrusion at the base of the control stem ruptured and ARs appeared. By contrast, ARs in OE lines occurred on day 3, 2 days earlier than in the control. Therefore, the *JrWOX11* gene is considered to promote the emergence of adventitious roots.

### Overexpression of *JrWOX11* influenced the development of the adventitious root system

**FIGURE 6:**
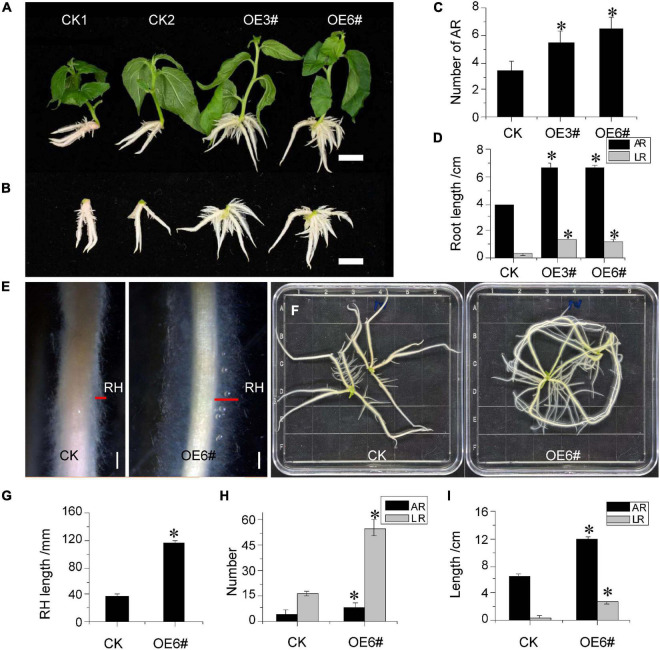
Overexpression of *JrWOX11* regulated root system architecture. **(A)** Comparison of AR number between CK and OE 3# and OE 3# 6# (2 weeks old). **(B)** The vertical view of ARs of CK and overexpressing lines 3# and 6# (2 weeks old). Bar = 1 cm. **(C,D)** Average AR length, AR number, and LR length in Control and the overexpressing lines (2 weeks old). **(E)** Root hairs of 4-week-old seedlings. Bars = 2 mm. **(F)** Root system phenotypes of the control group (CK) and WOX11 transgenic plants (OE#6) (4 weeks old). Bar = 1 cm. **(G–I)** Length of RH, average root length, and root number in Control and the overexpressing line (OE#6) (4 weeks old). Asterisk indicates a significant difference (*P < 0.05) between transgenic lines and WT plants.

To further verify whether JrWOX11 was involved in the development of the AR system, the root phenotypes of two OE lines (3# and 6#) were analyzed (Figures 6A–C). In 2-week-old seedlings, the AR number, length, and LR length were significantly higher in *JrWOX11*-transgenic lines (OE3# and OE6#) than controls (CK1 and CK2) (Figures 6H,I). Similarly, these root indices in 4-week-old seedlings of OE3# and OE6# lines were also significantly increased compared with controls (Figures 6E,F). Interestingly, transgenic plants overexpressing *JrWOX11* had significantly increased root hair length (Figures 6D,G). These results suggested that *JrWOX11* might regulate root development and root hair growth.

### *JrWOX11* increases tolerance to salt and osmotic stress

**FIGURE 7:**
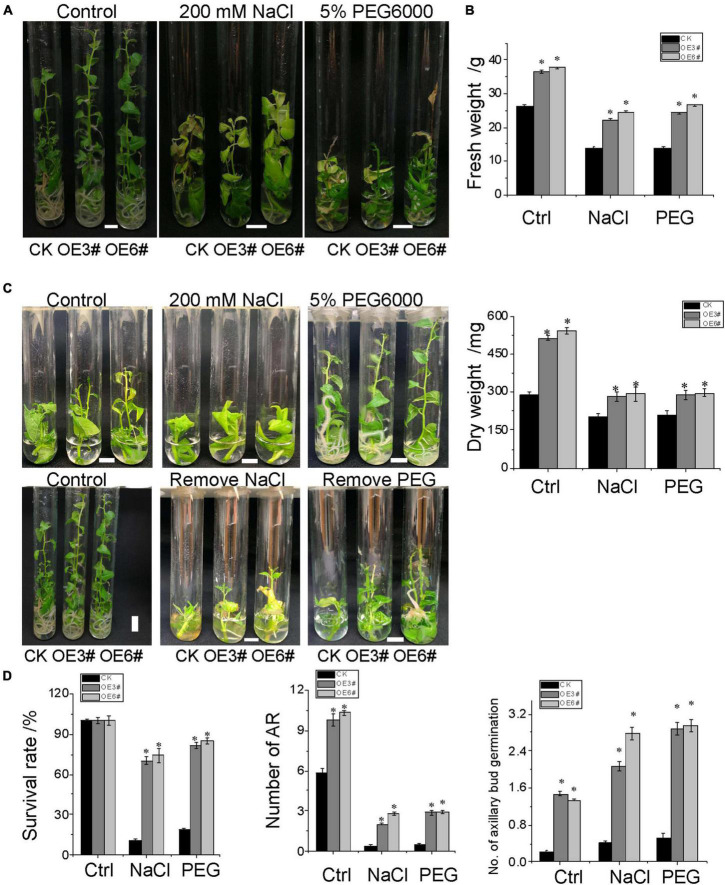
Stress response of *JrWOX11*-transgenic “84K” plants. **(A)** The growth and phenotype of *JrWOX11*-transgenic and WT poplar plants. **(B)** Effects of salt and osmotic stress on fresh weight and dry weight. Four-week-old seedlings were cultured vertically on the liquid RIM medium (1/2MS + 0.01 mg⋅L^– 1^ of IBA + 30 g⋅L^– 1^ of sucrose) supplemented with 200 mM NaCl or 5% PEG6000 for 2 weeks. **(C)** The photographs were taken, and the fresh weight and dry weight of each plant were measured. **(D)** The survival rate and regeneration response were observed when NaCl and PEG were removed. About 2.5 cm shoot cuttings were cultured vertically on liquid RIM medium supplemented with 200 mM NaCl or 5% PEG6000 for 4 weeks, then were transferred to liquid RIM medium without NaCl and PEG6000 for 2 weeks. The photographs were taken, the survival rate and the number of axillary bud regeneration, and the number of ARs of each plant were measured. All the above data were represented as the mean ± SD of at least three independent replications. Asterisk indicates a significant difference (*P < 0.05) between transgenic lines and WT plants.

To validate the effectiveness of root system architecture in NaCl and osmotic stress tolerance, 2-week-old OE (3# and 6#) and control (CK) seedlings were subjected to no-stress treatment (Control), 200 mM NaCl, and 5% w/v PEG6000 treatment in sterile seed tubes (Figures 7A,B). Three weeks later, the plant height, the aboveground fresh weight, and dry weight of OE3# and OE6# were superior to CK in the control treatment, suggesting that the overexpression of the *JrWOX11* gene improved the growth of transgenic plants. Under salt and osmotic stress, the aboveground fresh weight and dry weight were significantly increased in *JrWOX11*-overexpressing plants compared with CK plants, indicating that *JrWOX11* improved tolerance to NaCl and osmotic stress by promoting root growth.

Adventitious root formation is a *de novo* organogenesis process that enables plants to cope with environmental stresses. To evaluate the growth and root regeneration response of *JrWOX11*-overexpressing plants and control type (CK) to NaCl and osmotic stress, shoot cuttings were subjected to 200 mM NaCl and 5% w/v PEG6000 stress for 4 weeks. The root and aboveground biomass were significantly improved in *JrWOX11*-overexpressing plants compared with CK shoot cuttings under no-stress conditions (Figure 7C and Supplementray Figure 3). Additionally, the transgenic shoot cuttings showed significantly improved salt and osmotic tolerance compared to CK plants (Figure 7C). The survival rate, the number of axillary buds regenerated, and the number of ARs were increased substantially 2 weeks after plants were transferred to media without 200 mM NaCl and 5% w/v PEG6000 (Figure 7D). These findings suggested that *JrWOX11* enhanced tolerance to salt and osmotic stress by improving the *de novo* root organogenesis and shoot development (Liu J. et al., 2014, Liu B. et al., 2018; Hu and Xu, 2016; Zhou et al., 2017).

## Discussion

Recalcitrance to adventitious root formation from stem cuttings is a major limitation for clonal propagation in difficult-to-root trees (Macdonald, 1990). Given the energy and time required for maintaining difficult-to-root trees in a juvenile state in various environments, the development of rootstock varieties that are easy-to-root and tolerant to environmental stresses through molecular breeding is crucial. However, there is little knowledge about the genes conferring rooting capacity and abiotic stress tolerance. Previous studies have shown that the WOX11/12 gene, encoding a transcription factor, can be induced by salt and drought stress and that it participates in root formation and development in rice, *Arabidopsis*, and poplar (Zhao et al., 2009, 2015; Liu B. et al., 2014; Liu J. et al., 2014; Hu and Xu, 2016). Here, we isolated and characterized a *WOX11* gene in “ZNS” walnut, a difficult-to-root tree. In addition, in transgenic “84K” poplar overexpressing *JrWOX11*, the capacity to form adventitious roots and to develop the root system has been improved. Transgenic “84K” poplar overexpressing *JrWOX11* also showed increased NaCl and osmotic tolerance.

Previous studies have shown that *WOX11* is a crucial factor that controls normal cells shifting to root primordium cells (Liu J. et al., 2014). This process mainly depends on auxin biosynthesis and auxin transport (Sarkar et al., 2007; Chen et al., 2016; Wei et al., 2019). In *Arabidopsis*, AtWOX11 binds directly to the promoter of a LATERAL ORGAN BOUNDARIES DOMAIN transcription factor (AtLBD16) to result in the formation of LR root primordium cells (Wu et al., 2018). In the present study, overexpression of *JrWOX11* in “84K” poplar promoted the formation of root primordium cells 2s day earlier than in the control plants (Figure 5). It could be speculated that the *JrWOX11* gene regulated adventitious root formation *via* the auxin-dependent pathway.

Overexpression of *WOX11/12* genes, such as *OsWOX11*, *PtoWOX11*, *PeWOX11a*, *PeWOX11b*, and *PagWOX11/12*a resulted in increased numbers of adventitious and ectopic roots, which contributed to plant resistance to drought (Cheng et al., 2016; Wang et al., 2020). Furthermore, Wang et al. (2021) reported that *PagWOX11/12a* enhanced plant response to salt stress by controlling the redox metabolic pathways. Consistent with the previous studies in rice and poplar, *JrWOX11* not only increased the numbers of ARs, LRs, ectopic roots, and the length of RH but also improved the NaCl and osmotic tolerance in overexpressing “84K” poplar (Figure 7 and Supplementary Figure 3). Notwithstanding its limitations, the study suggests that *JrWOX11* increases “84K” poplar salt and osmotic tolerance by controlling adventitious root formation and root system development.

To investigate further the potential differences in *WOX11/12* between easy-to-root plants and difficult-to-root trees, we compared JrWOX11 to 26 WOX11/12 proteins from 15 species. In rice, *Arabidopsis*, and some easy-to-root trees, more than one WOX11/12 protein was found (Figure 1). However, “ZNS” walnut contained only one WOX11 protein, JrWOX11. In terms of protein distance matrix to JrWOX11, the descending order of similarity was: difficult-to-root trees (96.37 to 81.21%) > easy-to-root trees (74.44 to 61.48%) > *Arabidopsis* (57.01 and 54.67%) > rice (55.04 to 52.36%) > maize (49.78 and 45.22%). These results were consistent with the evolutionary relationships of the 27 WOX11/12 proteins analyzed. Furthermore, the MEME result of WOX11/12 pointed out another difference between easy-to-root trees and difficult-to-root trees, with motif10 structure (GGAIQY motif) lacking in difficult-to-root trees (Figure 1 and Supplementary Figure 2). Given that *JrWOX11* improves the capacity for adventitious root formation in transgenic “84K” poplar, a lack of the GGAIQY motif was not a determining factor for rooting difficulty in difficult-to-root trees. However, when the expression of *JrWOX11* was suppressed, the rooting capacity was significantly diminished (Chang et al., 2022). Age and associated sclerenchyma in difficult-to-root trees should also be considered (Sun and Zhu, 2020). It was reported that *WOX11/WOX12* could be suppressed by age-regulated *ETHYLENE INSENSITIVE 3* (*EIN3*) in *Arabidopsis* (Li et al., 2020). In walnut, rejuvenation diminished the extent of sclerenchyma, which is beneficial to auxin transport and rooting (Liu H. et al., 2018).

We also analyzed the differences in the *cis*-element of *WOX11/12* genes between easy-to-root trees and difficult-to-root trees. Compared with OsWOX11, OsWOX12a, OsWOX12b, AtWOX11, AtWOX12, PtrWOX11/12a, and PtrWOX11/12b, the promoter of *JrWOX11* in “ZNS” walnut and *J. regia* (Chang et al., 2020) harbored more AuxRE and ABREs but fewer MBS, G-box, W-box, and DRE motifs (Figure 2 and Supplementary Figure 4), which are generally known as elements that respond to abiotic stresses (Shi et al., 2011; Lee et al., 2013; Baruah et al., 2021). The result might imply that *JrWOX11* enhanced the stress tolerance of transgenic “84K” poplar *via* the ABA-dependent pathways (Putterill et al., 2021). Further work is needed to test this suggestion.

## Conclusion

In the present study, we successfully cloned full-length cDNA and genomic sequences of *JrWOX11* from “ZNS” walnut; its expression was induced by ABA, NaCl, and PEG treatment. Compared to easy-to-root plants, walnut harbored fewer *WOX11/12* genes, the JrWOX11 protein sequence lacked the “GGAIKY” motif, and the promoter of JrWOX11 harbored more AuxRE and ABREs but fewer MBS, G-box, W-box, and DRE motifs. Additionally, the overexpression of *JrWOX11* significantly improved the adventitious root formation capacity, increased the number of AR and LR, and elongated the LR and RH lengths in “84K” poplar overexpressing *JrWOX11*. Moreover, the tolerance to NaCl and osmotic stress in transgenic “84K” poplar plants and shoot cuttings was also enhanced. This likely reflects the fact that *JrWOX11* promotes *de novo* root organogenesis and root system development, thereby regulating NaCl and osmotic tolerance. The differences in WOX11/12 between easy-to-root plants and difficult-to-root trees provide the foundation for the molecular breeding of difficult-to-root trees.

## Data availability statement

The data presented in this study are deposited in the GenBank repository, accession number: ON979687.

## Author contributions

DP and QZ contributed substantially to the experimental design, conceived the study, and approved the final manuscript. XS contributed to funding, interpreted the data, authored or reviewed the draft, and revised the manuscript. ML gave substantial suggestions to the manuscript. YC, PZ, and XL carried out mainly experiments, finished the draft of the manuscript, and comprehensively analyzed the data from all experimental results. All authors contributed to the article and approved the submitted version.

## Footnotes

1
http://meme-suite.org/tools/meme

2
http://gsds.cbi.pku.edu.cn/

3
http://bioinformatics.psb.ugent.be/webtools/plantcare/html/

## Supplementary material

Supplementary Figure 1 The detection of transgenic 84K poplar. **(a)** DNA detection of *HPT* gene in WT and transgenic line OE1#∼OE14 #. **(b)** The expression of the *JrWOX11* gene in stems of WT and transgenic line OE1#∼OE14 #5 microshoots.

Supplementary Figure 2 Sequence alignment of homologous WOX11/12 proteins. **(a)** Phylogenetic tree based on full-length coding sequences of WOX11/12 genes using the NJ method. **(b)** Motifs analysis of WOX11/12 proteins. MEME software was used to search motifs, and then redraw the map with the software TBtools **(c)**. *Zm*, *Zea mays*; *Os*, *Oryza sativa*; *At*, *Arabidopsis thaliana*; *Ptr*, *Populus trichocarpa*; *Pto*, *Populus tomentosa*; *Pe*, *P. deltoides* × *P. euramericana* cv. “Nan-lin895,” *Pag*, *P. alba* × *P. glandulosa* cv. “84K,” *Sp*, *Salix purpurea; Md*, *Malus domestica; Mn*, *Morus notabilis; Dz*, *Durio zibethinus; Jr, Juglans hindsii* × *J. regia* cv. “ZNS,” *Ci, Carya illinoinensis; Qs*, *Quercus suber; Ql*, *Quercus lobuta; Cm*, *Castanea mollissima*.

Supplementary Figure 3 Plant phenotype determination during NaCl and PEG treatment. **(a)** Shoot cuttings of CK, OE3#, and OE6 # were cultured vertically on the liquid RIM medium (1/2MS + 0.01 mg⋅L^–1^ of IBA + 30 g⋅L^–1^ of sucrose) as Control. **(b,c)** Shoot cuttings of CK, OE3#, and OE6 were cultured vertically on liquid RIM medium supplemented with 100 and 200 mM NaCl. **(d,e)** Shoot cuttings of CK, OE3#, and OE6 were cultured vertically on liquid RIM medium supplemented with 5 and 10% PEG6000. Photographic pictures were made once a week. 1∼4 w: 1∼4 weeks after the shoot cuttings were cultured on the corresponding medium. R1W∼R2W: 1∼4 weeks after the corresponding stress is removed.

Supplementary Figure 4 Promoter sequence alignment of *WOX11* gene in “ZNS” walnut and *Juglans regia*.
