# Supplementary figures and images for: Characterization of walnut JrWOX11 and its overexpression provide insights into adventitious root formation and development and abiotic stress tolerance
Source: Front Plant Sci. 2022 Sep 6;13:951737. doi: 10.3389/fpls.2022.951737 (PMC9485816; doi:10.3389/fpls.2022.951737)

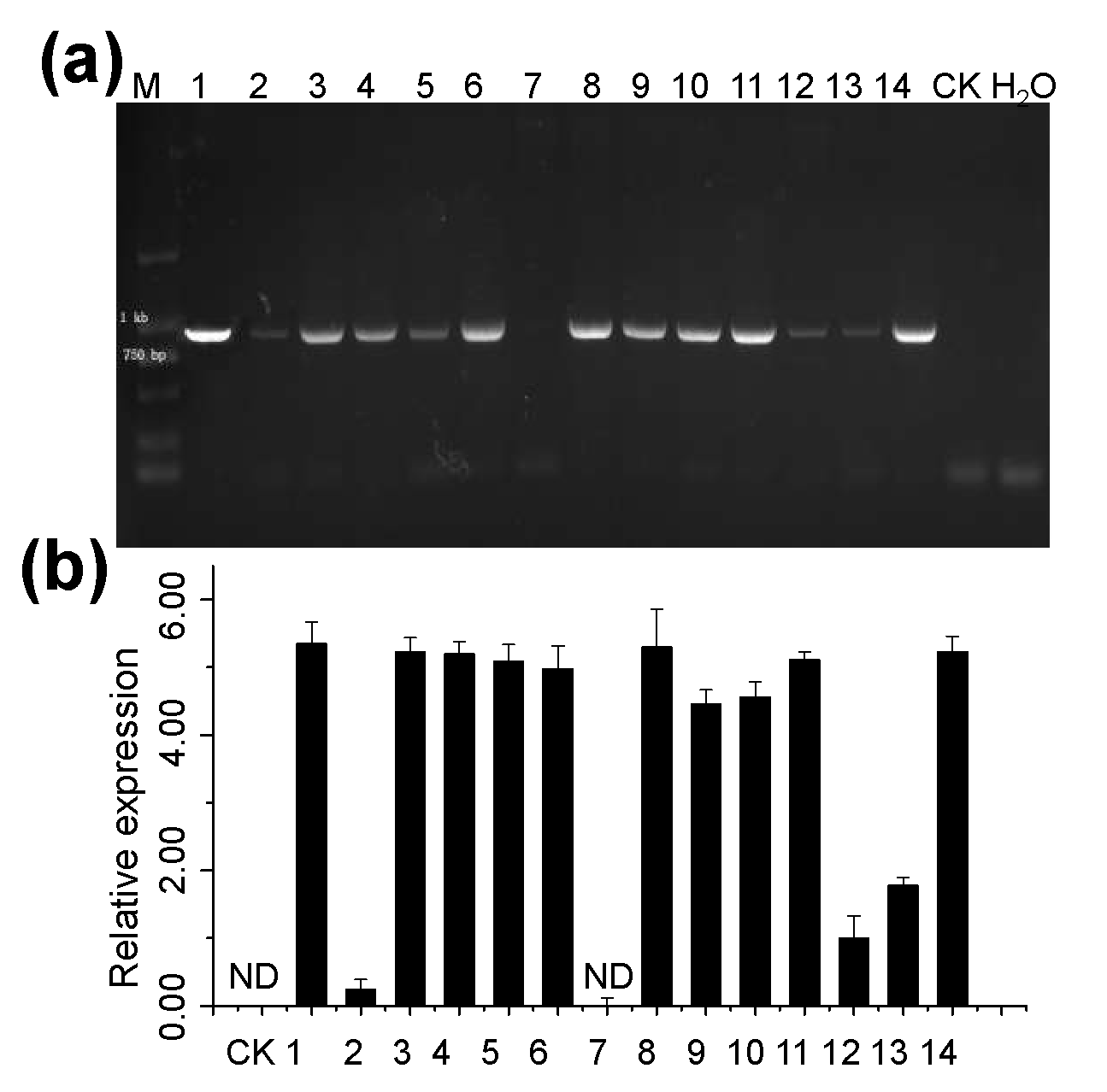

Supplement: Supplementary Figure 1 — The detection of transgenic 84K poplar. (a) DNA detection of HPT gene in WT and transgenic line OE1#∼OE14 #. (b) The expression of the JrWOX11 gene in stems of WT and transgenic line OE1#∼OE14 #5 microshoots. [file Image_1.TIF]

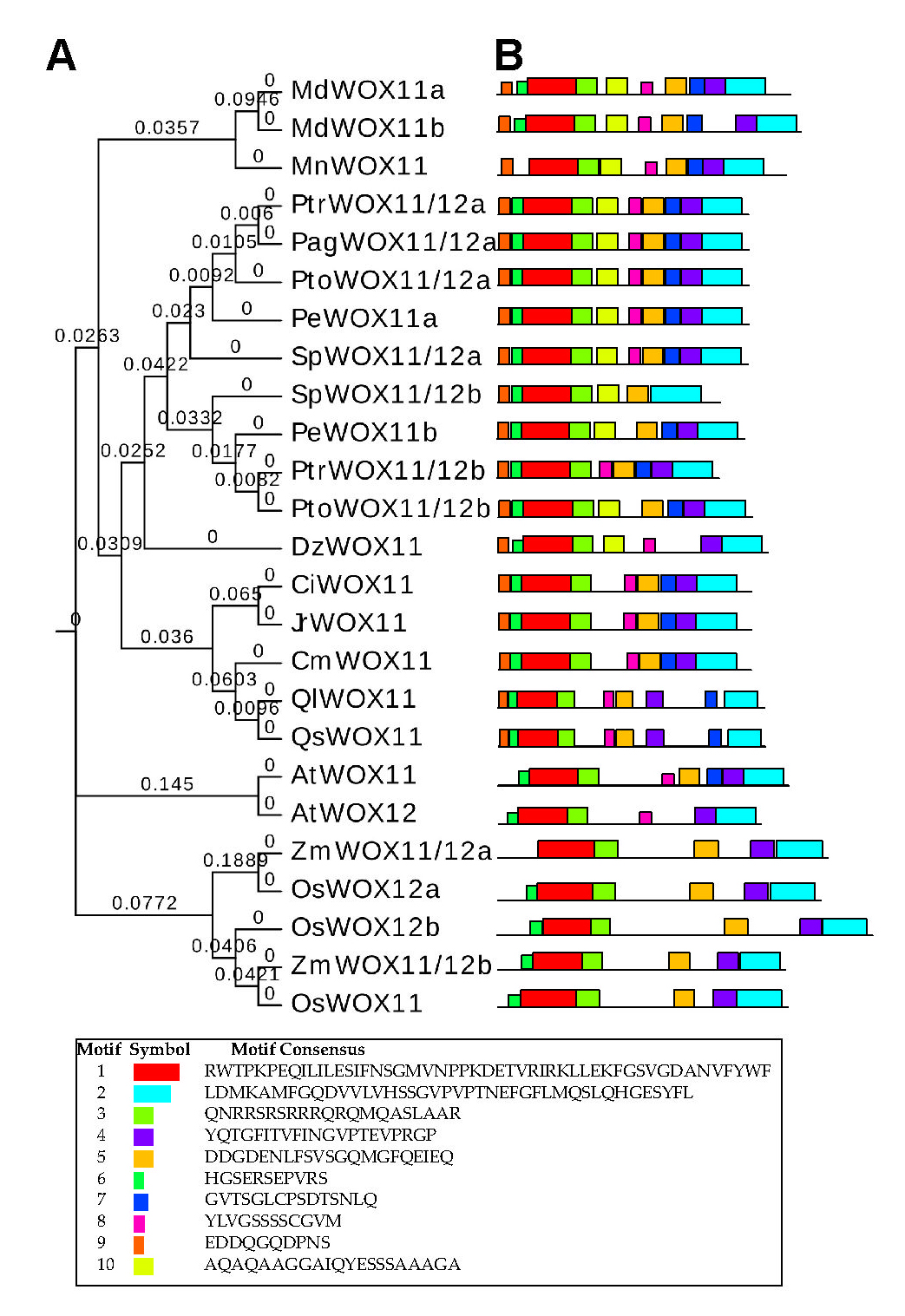

Supplement: Supplementary Figure 2 — Sequence alignment of homologous WOX11/12 proteins. (a) Phylogenetic tree based on full-length coding sequences of WOX11/12 genes using the NJ method. (b) Motifs analysis of WOX11/12 proteins. MEME software was used to search motifs, and then redraw the map with the software TBtools (c). Zm, Zea mays; Os, Oryza sativa; At, Arabidopsis thaliana; Ptr, Populus trichocarpa; Pto, Populus tomentosa; Pe, P. deltoides × P. euramericana cv. “Nan-lin895,” Pag, P. alba × P. glandulosa cv. “84K,” Sp, Salix purpurea; Md, Malus domestica; Mn, Morus notabilis; Dz, Durio zibethinus; Jr, Juglans hindsii × J. regia cv. “ZNS,” Ci, Carya illinoinensis; Qs, Quercus suber; Ql, Quercus lobuta; Cm, Castanea mollissima. [file Image_2.tif]

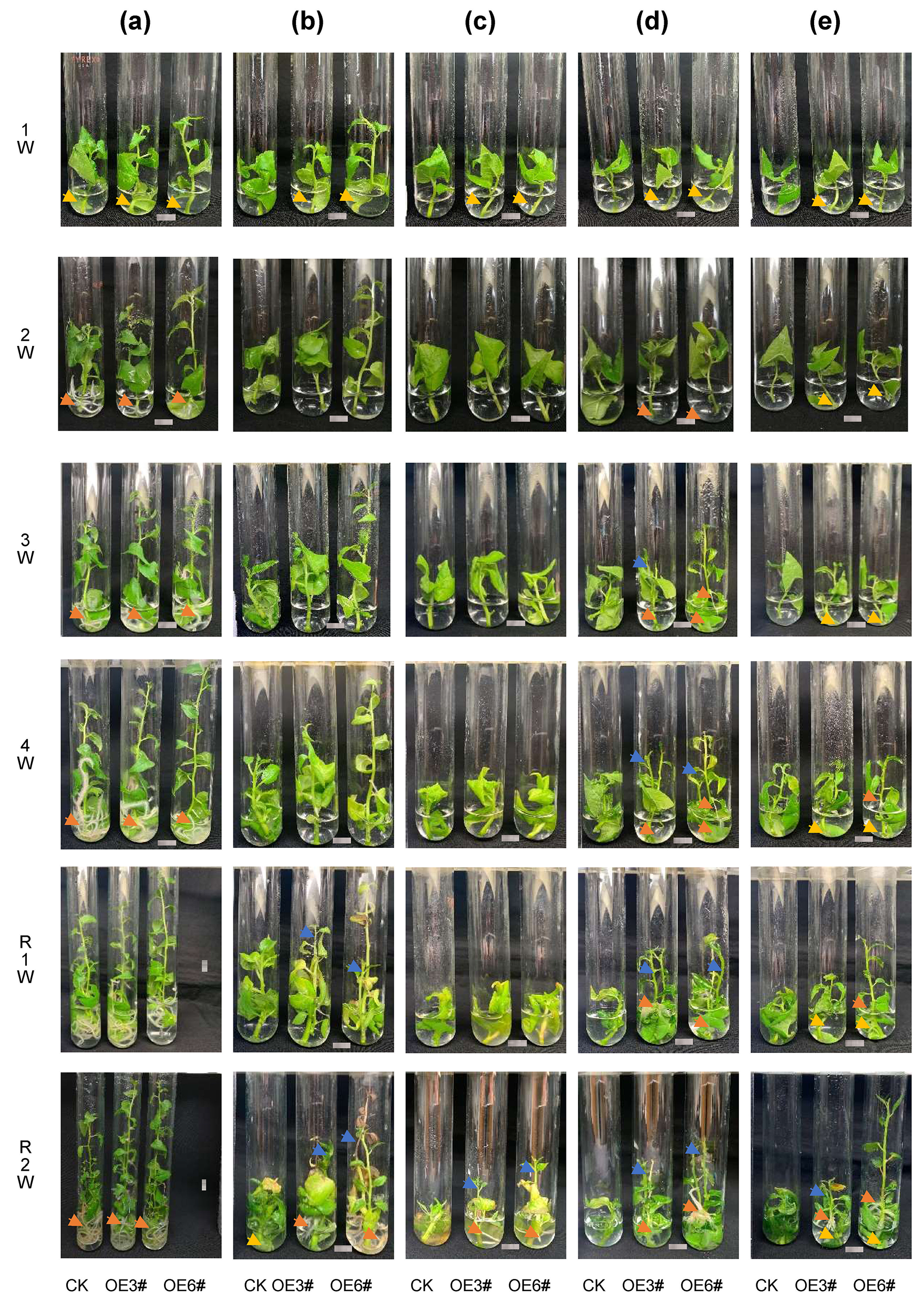

Supplement: Supplementary Figure 3 — Plant phenotype determination during NaCl and PEG treatment. (a) Shoot cuttings of CK, OE3#, and OE6 # were cultured vertically on the liquid RIM medium (1/2MS + 0.01 mg⋅L–1 of IBA + 30 g⋅L–1 of sucrose) as Control. (b,c) Shoot cuttings of CK, OE3#, and OE6 were cultured vertically on liquid RIM medium supplemented with 100 and 200 mM NaCl. (d,e) Shoot cuttings of CK, OE3#, and OE6 were cultured vertically on liquid RIM medium supplemented with 5 and 10% PEG6000. Photographic pictures were made once a week. 1∼4 w: 1∼4 weeks after the shoot cuttings were cultured on the corresponding medium. R1W∼R2W: 1∼4 weeks after the corresponding stress is removed. [file Image_3.TIF]

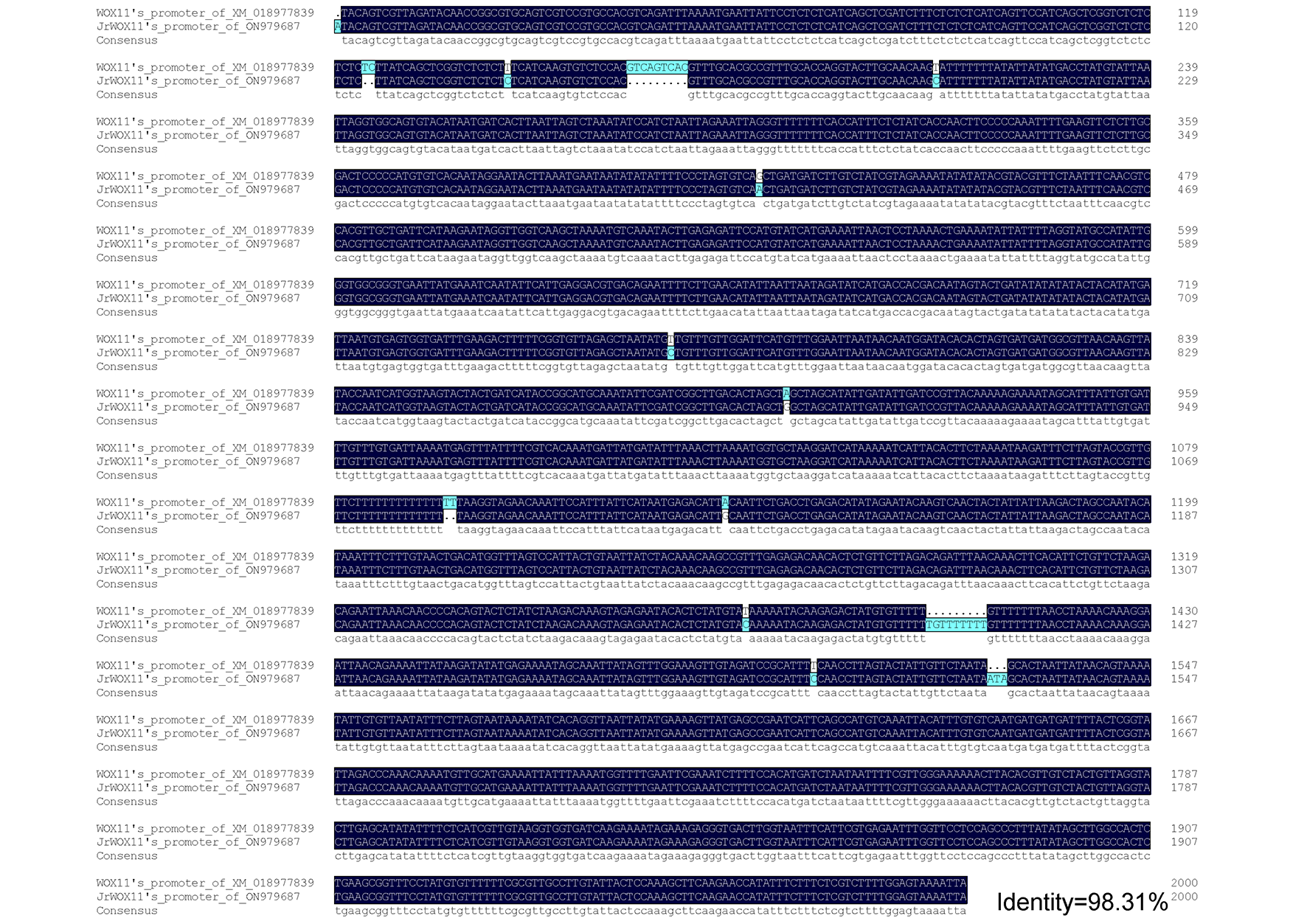

Supplement: Supplementary Figure 4 — Promoter sequence alignment of WOX11 gene in “ZNS” walnut and Juglans regia. [file Image_4.TIF]
