# Supplementary material for: Characterization of walnut JrWOX11 and its overexpression provide insights into adventitious root formation and development and abiotic stress tolerance
Source: Front Plant Sci. 2022 Sep 6;13:951737. doi: 10.3389/fpls.2022.951737 (PMC9485816; doi:10.3389/fpls.2022.951737)
Supplement: Supplementary file 5 [file Data_Sheet_1.docx]

Characterization and overexpression of *JrWOX11* from walnut provide insight into adventitious root formation, development and abiotic stress tolerance

Yingying Chang^1,2†^, Xiaobo Song^2†^, Mingjun Li^1^, Qixiang Zhang^3^, Pu Zhang^2^, Xiashuo Lei^2^, Dong Pei^2*^

1. Engineering Laboratory of Green Medicinal Material Biotechnology of Henan Province, Engineering Technology Research Center of Nursing and Utilization of Genuine Chinese Crude Drugs of Henan Province, College of Life Science, Henan Normal University, Xinxiang 453007, China

2. State Key Laboratory of Tree Genetics and Breeding, Key Laboratory of Tree Breeding and Cultivation of the State Forestry and Grassland Administration, Research Institute of Forestry, Chinese Academy of Forestry, Beijing 100091, China

3. The Nurturing Station for the State Key Laboratory of Subtropical Silviculture, School of Forestry and Biotechnology, Zhejiang A&F University, Lin’an 311300, China

^†^ These two authors contributed to this work equally.

*Corresponding author: Dong Pei, E-mail: pei.dong@caf.ac.cn

Supplemental Tables

Supplemental Table 1. The primer sequences used in this research

| **Primer** | **Primer sequence（F: 5'→3'）** | **Application** |
| --- | --- | --- |
| *qJrWOX11-F* | GACGTAGTGTTGGTCCATTGCT | qRT-PCR |
| *qJrWOX11-R* | GCCCTCTTTCTTCCCCTTTAA |  |
| *GAPDH-F* | ATGATGTCAAGGAAGGACTC | qRT-PCR |
| *GAPDH-R* | CACAATGATCTCAGCTCCG |  |
| *JrWOX11-F* | ggggacaactttgtacaaaaaagttggaTGGAAGAACAAGGGCA | Gene cloning |
| *JrWOX11-R* | ggcggccgcacaactttgtacaagaaagttgggtaCTAGAGTGAGCCCTCTTTCT |  |
| *HPT-F* | GGTTTCCACTATCGGCGAGT | Transgenetic plant detection |
| *HPT-R* | TAAATAGCTGCGCCGATGGT |  |
| *proJrWOX11-F* | cggggatcctctagagtcgacAGTCGTTAGATACAACCGGCG | Cloning promoter |
| *proJrWOX11-R* | ttaccctcagatctaccatggTTGGAGTAATACAAGGCAACGC |  |

Note: The sequences marked with single underline were the joint sequence for gateway kit.

Supplemental Table 2. Accession numbers and basic characteristics of the WOX11/12 proteins

| Gene | Gene ID | Protein Length/aa | Molecular Weight  (Mw)/Da | Isoelectric Point (pI) | Protein  Hydrophobicity | Aliphatic Amino Acid Index | Similarity to JrWOX11  (%) |
| --- | --- | --- | --- | --- | --- | --- | --- |
| *ZmWOX11/12a* | NP_001147238.1 | 294 | 31473.19 | 8.50 | -0.360 | 59.62 | 45.22 |
| *ZmWOX11/12b* | XP_008653490.1 | 258 | 27449.68 | 7.02 | -0.324 | 64.03 | 49.78 |
| *OsWOX11* | XP_015645719.1 | 262 | 27587.79 | 7.95 | -0.313 | 70.82 | 55.04 |
| *OsWOX12a* | XP_015649642.1 | 284 | 30271.64 | 6.34 | -0.373 | 66.76 | 52.97 |
| *OsWOX12b* | XP_015629005.1 | 356 | 36474.80 | 8.67 | -0.115 | 65.93 | 52.36 |
| *AtWOX11* | NP_001118563.1 | 297 | 32130.32 | 6.48 | -0.533 | 54.18 | 54.67 |
| *AtWOX12* | NP_001190327.1 | 268 | 29713.91 | 6.09 | -5.579 | 58.25 | 57.01 |
| *PtrWOX11/12a* | Potri.019G040800 | 254 | 28020.10 | 5.69 | -0.454 | 68.62 | 66.53 |
| *PtrWOX11/12b* | Potri.019G040800 | 248 | 27466.51 | 6.16 | -0.452 | 66.68 | 65.27 |
| *PtoWOX11/12a* | AHL29320.1 | 255 | 28013.00 | 5.50 | -0.461 | 66.47 | 61.48 |
| *PtoWOX11/12b* | AHL29321.1 | 249 | 27504.52 | 6.30 | -0.465 | 66.43 | 65.33 |
| *PeWOX11/12a* | (Xu et al., 2015) | 254 | 28039.03 | 5.87 | -0.536 | 64.02 | 70.17 |
| *PeWOX11/12b* | (Xu et al., 2015) | 248 | 27346.37 | 6.16 | -0.475 | 68.27 | 74.66 |
| *PagWOX11/12a* | (Wang et al., 2020) | 255 | 28084.18 | 5.69 | -0.407 | 70.27 | 69.87 |
| *SpWOX11/12a* | SapurV1A.1660s0010 | 254 | 27933.90 | 5.87 | -0.535 | 64.41 | 67.90 |
| *SpWOX11/12b* | SapurV1A.0631s0110 | 236 | 25952.75 | 5.68 | -0.547 | 63.52 | 68.03 |
| *MdWOX11a* | XP_017191887.1 | 276 | 30161.64 | 6.04 | -0.389 | 74.46 | 69.96 |
| *MdWOX11b* | XP_008391727.1 | 279 | 30496.87 | 5.65 | -0.474 | 68.46 | 70.08 |
| *MnWOX11* | EXC36166.1 | 295 | 32770.62 | 6.89 | -0.550 | 62.75 | 69.26 |
| *DzWOX11* | XP_022743818.1 | 262 | 28486.12 | 6.38 | -0.324 | 69.50 | 69.20 |
| *QlWOX11* | XP_030925492.1 | 253 | 27821.09 | 5.60 | -0.392 | 70.04 | 82.57 |
| *QsWOX11* | XP_023880720.1 | 253 | 27705.89 | 5.44 | -0.411 | 68.10 | 81.82 |
| *CmWOX11* | KAF3972145.1 | 252 | 27692.01 | 5.67 | -0.373 | 69.92 | 82.23 |
| *CiWOX11* | XP_042943179.1 | 248 | 27114.22 | 5.56 | -0.414 | 68.71 | 96.37 |
| *JrWOX11* | ON979687 | 248 | 27058.19 | 5.42 | -0.402 | 70.69 | 100 |

Note: *Zm*: *Zea mays*, *Os*: *Oryza sativa*, *At*: *Arabidopsis thaliana*, *Ptr*: *Populus trichocarpa*, *Pto*: *Populus tomentosa*, *Pe*: *P. deltoides* ×*P. euramericana* cv. ‘Nan- lin895’, *Pag*: *P.* *alba* × *P. glandulosa* cv. ‘84K’, *Sp*: *Salix purpurea, Md*: *Malus domestica, Mn*: *Morus notabilis, Dz*: *Durio zibethinus, and Jr:* *Juglans hindsii ×J. regia* cv. ‘ZNS’, *Ci: Carya illinoinensis, Qs*: *Quercus suber, Ql*: *Quercus lobuta, Cm*: *Castanea mollissima*.

Supplemental Table 3. Cis-regulatory elements predicted in promoter region of *WOX11/12* Homologous genes

| *cis*-Elements | Sequence | Predicted funciton | *OsWOX11* | *OsWOX12a* | *OsWOX12b* | *AtWOX11* | *AtWOX12* | *PtrWOX11/12a* | *PtrWOX11/12b* | *JrWOX11* | Classification |
| --- | --- | --- | --- | --- | --- | --- | --- | --- | --- | --- | --- |
| OSE1ROOTNODOULE | AAAGAT | Root specificity element | 3 | 1 | 2 | 5 | 5 | 5 | 3 | 2 | Root specificity |
|  |  |  | -6(+) | -1731(-) | -1903(+) | -107(+) | -496(+) | -1582(+) | -174(+) | -264(-) |  |
|  |  |  | -771(-) |  | -1333(-) | -113(+) | -998(+) | -1915(+) | -534(+) | -1905(-) |  |
|  |  |  | -912(-) |  |  | -424(+) | -608(-) | -227(-) | -871(+) |  |  |
|  |  |  |  |  |  | -1024(+) | -1174(-) | -1092(-) |  |  |  |
|  |  |  |  |  |  | -1800(-) | -1601(-) | -1440(-) |  |  |  |
| TGA-element | AACGAC | Auxin-responsive element | 0 | 1 | 2 | 0 | 0 | 0 | 0 | 1 | Plant hormone |
|  |  |  |  | -1621(+) | -934(+) |  |  |  |  | -1982(-) |  |
|  |  |  |  |  | -1382(+) |  |  |  |  |  |  |
| AuxRE | GAGAC/TGTATC | Auxin-responsive element | 2 | 4 | 2 | 5 | 3 | 2 | 3 | 10 |  |
|  |  |  | -992(+) | -651(+) | -532(+) | -200(+) | -1604(+) | -1901(+) | -1304(+) | -596(+) |  |
|  |  |  | -1018(+) | -176(-) | -1487(+) | -1353(+) | -910(-) | -1987(-) | -1421(+) | -722(+) |  |
|  |  |  |  | -243(-) |  | -486(-) | -1246(-) |  | -1327(-) | -837(+) |  |
|  |  |  |  | -565(-) |  | -638(-) |  |  |  | -859(+) |  |
|  |  |  |  |  |  | -1444(-) |  |  |  | -1444(+) |  |
|  |  |  |  |  |  |  |  |  |  | -1181(-) |  |
|  |  |  |  |  |  |  |  |  |  | -1832(-) |  |
|  |  |  |  |  |  |  |  |  |  | -1850(-) |  |
|  |  |  |  |  |  |  |  |  |  | -1874(-) |  |
|  |  |  |  |  |  |  |  |  |  | -1975(-) |  |
| ARR1AT | NAGATT | Cytokinin response factor ARR1 regulatory site | 4 | 1 | 5 | 8 | 9 | 8 | 8 | 5 |  |
|  |  |  | -4(+) | -270(+) | -503(+) | -422(+) | -713(+) | -147(+) | -172(+) | -265(+) |  |
|  |  |  | -1142(+) |  | -1788(+) | -464(+) | -1235(+) | -1252(+) | -389(+) | -699(+) |  |
|  |  |  | -1792(+) |  | -556(-) | -530(+) | -1618(+) | -1580(+) | -568(+) | -926(+) |  |
|  |  |  | -1084(-) |  | -967(-) | -508(+) | -1992(+) | -1913(+) | -869(+) | -1452(+) |  |
|  |  |  |  |  | -1334(-) | -1435(+) | -23(-) | -228(-) | -1003(+) | -1941(+) |  |
|  |  |  |  |  |  | -1419(-) | -60(-) | -1093(-) | -1718(+) |  |  |
|  |  |  |  |  |  | -1763(-) | -569(-) | -1397(-) | -1785(+) |  |  |
|  |  |  |  |  |  | -1938(-) | -609(-) | -1616(-) | -511(-) |  |  |
|  |  |  |  |  |  |  | -645(-) |  |  |  |  |
| GARE-motif | TAACAAR | Gibberellin responsive element | 1 | 1 |  | 1 | 3 | 1 | 0 | 2 |  |
|  |  |  | -1272(+) | -989(-) |  | -1432(-) | -224(-) | -253(-) |  | -694(+) |  |
|  |  |  |  |  |  |  | -233(-) |  |  | -1157(+) |  |
|  |  |  |  |  |  |  | -1856(-) |  |  |  |  |
| ABRE | ACGTG | Abscisic acid responsive element | 2 | 2 | 0 | 0 | 3 | 1 | 2 | 7 |  |
|  |  |  | -504(+) | -691(-) |  |  | -167(+) | -1983(+) | -97(-) | -1350(+) |  |
|  |  |  | -1218(+) | -1392(+) |  |  | -1089(+) |  | -1323(-) | -220(-) |  |
|  |  |  |  |  |  |  | -1311(-) |  |  | -1507(-) |  |
|  |  |  |  |  |  |  |  |  |  | -1532(-) |  |
|  |  |  |  |  |  |  |  |  |  | -1818(-) |  |
|  |  |  |  |  |  |  |  |  |  | -1827(-) |  |
|  |  |  |  |  |  |  |  |  |  | -1947(-) |  |
| DRE | ACCGAGA | Ethylene/salt responsive element | 0 | 0 | 0 | 0 | 0 | 1 | 0 | 0 | Abiotic stress response |
|  |  |  |  |  |  |  |  | -310(-) |  |  |  |
| MBS | CAACTG | Drought responsive element |  |  | 1 | 1 | 0 | 0 | 0 | 0 |  |
|  |  |  |  |  | -59(+) | -1379(-) |  |  |  |  |  |
| W-box | TTGACC | Drought responsive element |  | 1 | 2 | 3 | 0 | 2 | 2 | 1 |  |
|  |  |  |  | -530(-) | -290(-) | -1539(+) |  | -1248(+) | -1021(+) | -1480(-) |  |
|  |  |  |  |  | -422(+) | -261(-) |  | -1688(+) | -1161(+) |  |  |
|  |  |  |  |  |  | -1961(-) |  |  |  |  |  |
| WOX-consensus | TTAATGG/C | 0 | 0 | 2 | 0 | 0 | 1 | 0 | 2 | 0 | WOX11 binding site |
|  |  |  |  | -189(+) |  |  | -1142(+) |  | -1037(-) |  |  |
|  |  |  |  | -1759(+) |  |  |  |  | -1500(-) |  |  |
